# Non-Steroidal Biphenyl Gelators: Correlation of Xerogel Structure with Solid-State Structure and Circular Dichroism Spectroscopy

**DOI:** 10.3390/gels4020034

**Published:** 2018-04-16

**Authors:** H. Cristina Geiger, David K. Geiger, William R. Roberts, Dominic L. Morell, Paul Huttunen, Jennifer L. Schulman, Melanie Tran, Dori Farthing

**Affiliations:** 1 Department of Chemistry, State University of New York College at Geneseo, Geneseo, NY 14454, USA; geiger@geneseo.edu (D.K.G.); wrroberts95@gmail.com (W.R.R.); dominicmorell@gmail.com (D.L.M.); phkalevi@gmail.com (P.H.); jls67@geneseo.edu (J.L.S.); mt22@geneseo.edu (M.T.); 2 Department of Geological Science, State University of New York College at Geneseo, Geneseo, NY 14454, USA; farthing@geneseo.edu

**Keywords:** organogels, XRD, SEM, CD, gelation

## Abstract

Because the factors favoring the formation of well-formed single crystals are dissimilar to those conducive to gel formation, few examples of single-crystal structural characterizations of organogelators are found in the literature. A series of biphenyl methyl and ethyl diester derivatives of varying chain length were synthesized and their gelation abilities explored. X-ray diffraction of single crystals of one of the gelators reveals a columnar extended structure. Based on XRD results for xerogels obtained from the reported organogelators, the members of the series are isostructural and so also adopt a columnar superstructure. Scanning electron microscopy (SEM) was used for the investigation of the morphology of the xerogels, which display either platelet-like morphologies or more typical entangled twisted ribbon-like aggregates. The gels exhibit chirality, which depends on the sol-gel transition history, as observed by induced circular dichroism (ICD) spectroscopy.

## 1. Introduction

The study of molecular gels continues to be of much interest in the field of soft matter science as observed by the large number of citations reported in the literature [1]. Although much has been reported about the structure and properties of organogels, many questions still remain, including the relationship between gelator structure in the gel and in the solid state, the state or states of the solvent, the factors that determine in which solvents a given gelator will gel and how the material properties of the gel on a macroscopic scale are influenced by the nano- and meso-structures of the gel [2,3,4,5,6]. Although serendipity plays a large role in the discovery of new gelators, new approaches, especially those applying state-of-the-art computational techniques such as the Kitaigorodskii–Aufbau principle (KAP) [7,8], density functional theory (DFT), Monte Carlo simulation and other types of calculations can be used to explore the connections between the structure of gelators at an early stage of aggregation and the stability of a gel as observed experimentally [9]. 

The large number of reported studies is due in part to the wide range of gel applications. Gels find applications in drug delivery [10,11,12], environmental spill-oil recovery [13], nanomaterials [14] and organic solar cells [15]. Zhou and co-workers recently reported a series of two-component organogelators consisting of a triamide moiety and three trans-azobenzene groups located on the edge of the molecule [16]. These gelators are able to form photoactive organogels, which exhibit photo-induced structural changes, rendering them applicable to use in coatings and other “intelligent” materials.

Organogels are obtained by dissolving a small amount of a low-molecular-mass organic gelator in an organic solvent. Gel formation requires that the dissolved gelator self-assemble into a three-dimensional network structure incorporating solvent via non-covalent interactions rather than self-assembly followed by crystallization. Previous reports have explored the role of weak C-H/π interactions [17], the effects of van der Waals forces among stacked cholesterol units [18,19] and the importance of hydrogen bonding between gelator molecules [20] in the gelation process.

As reported, experimental factors like rates of heating and cooling (altering the gel/sol equilibrium) show drastic effects on the supramolecular structure of organogels [1,21]. To minimize the effects of the process referred to as Ostwald ripening, which refers to the susceptibility of the sol-to-gel transition to hysteresis effects, rate of heating and history of the gel [22,23], a demountable cell was employed in the spectroscopy studies reported herein to minimize these effects, as the gel is formed within a sealed cell without physical disruption.

Among the types of effective low molecular mass organic gelators (LMOGs) found in different investigations are Aromatic Linker Steroid (ALS), and A(LS)_2_, where A is an aromatic moiety, L a methylene chain linker and S a steroidal unit. Of these, the cholesterol-based LMOGs have attracted a considerable amount of attention because of their versatility in gelation and numerous structural variations. The cholesteryl moiety provides extra stability to the gels by stacking in a helical fashion due to its strong lipophilic character, which tends to self-assemble in polar solvents [19,24].

In a previous study [18], we reported the gelation properties of three A(LS)_2_-type gelators where A is a biphenyl, L a four and eight chain linker and S a cholesteryl moiety. These molecules produced thermoreversible chiral stable gels in n-octanol and n-butanol. We attributed the stacking of the cholesteryl units as one of the major driving forces for the stability and chirality of these gels. To further explore the role of the cholesteryl moiety on the supramolecular aggregation of this type of gel, we synthesized similar gelator molecules by substituting the cholesteryl ester moiety for simple methyl and ethyl groups. Surprisingly, the alkyl biphenyl ester derivatives form strong, chiral gels similar to the cholesteryl homologs. We report on the synthesis and characterization of six, novel LMOGs, BBOn-Me and BBOn-Et (*n* = 6, 8, 10). Gel aggregation was examined by ultraviolet-visible (UV-Vis), emission and induced circular dichroism (ICD) spectroscopies. In addition, xerogels obtained from the organogels were examined by SEM and XRD.

## 2. Results and Discussion

### 2.1. Gelation Studies

Gelation studies were conducted in eight different solvents as shown in Table 1. The compounds with the shortest alkyl chain, **1** and **2**, exhibit the weakest gelation abilities. Compounds with the longest alkyl chain, **5** and **6**, were stronger gelators in the range of solvents tested. This observation is consistent with other reported studies where variation in the structure of the linkers of gelator molecules alters their aggregation and gelation ability [25,26]. n-Octanol proved to be the best solvent for gelation as the strongest gels were formed with almost all gelators (Compounds **1**, **3**–**6**).

### 2.2. Phase Transition Temperature (T_g_)

The gel-sol phase transition temperatures of the gels obtained from the methyl ester gelators are higher than the ones obtained from the analogous ethyl ester gelators, and the T_g_ increases as the alkyl chain length increases (Table 2). Although the methyl and ethyl ester biphenyl gelators form stable and reversible gels in n-octanol, the T_g_ of BBO8-Me and BBO8-Et are significantly lower than the T_g_ of BBO8-chol gel, where the gelator consists of a cholesteryl moiety instead of a small alkyl group [18]. These results are in agreement with the observation that cholesteryl derivatives have a strong tendency to self-aggregate due to van der Waals interactions, but our results show that the interactions between the linkers are also an important contributor to the formation and stability of the gel.

### 2.3. Spectroscopic Studies

Absorption and emission spectra were used to monitor the interactions between the biphenyl rings in order to evaluate aggregation during gel formation. Diluted 1 × 10^−5^ M octanol solutions of all gelators show an absorption maximum at a wavelength of 265 nm. As expected, each solution exhibits a nearly identical absorption spectrum since their structures consist of the same chromophore. A very slight red shift (1–2 nm) and broader absorption spectra are observed with the 1.5% (*w*/*w*) n-octanol gels.

**Figure 1:**
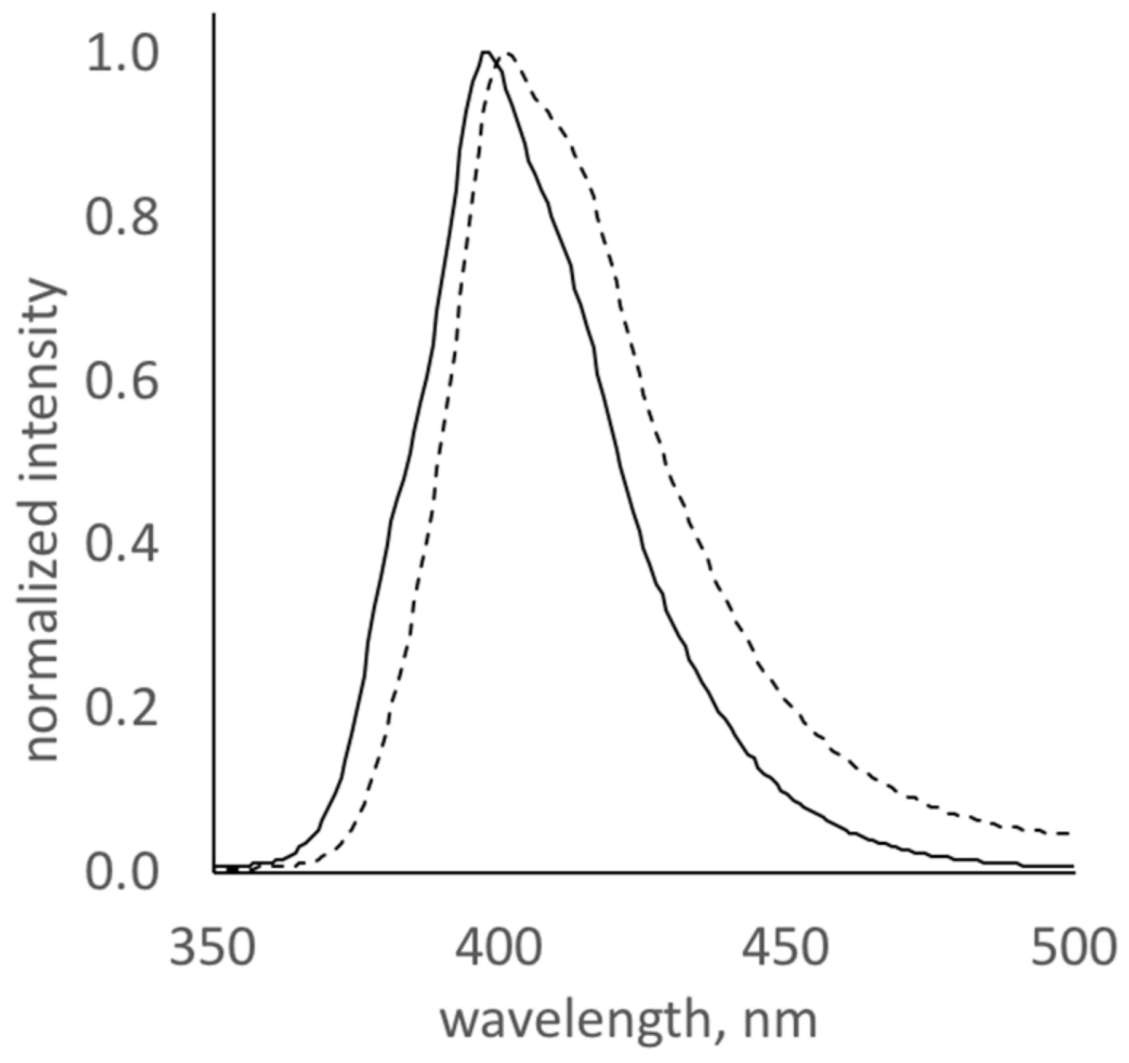
Normalized emission spectra of BBO8-Me in 10^−5^ M n-octanol solution (solid) and 1.5% (*w*/*w*) n-octanol gel (dashed) excited at 270 nm.

To investigate aggregate formation in the gel phase, emission studies were performed in both the gel form (1.5%, *w*/*w*) and in diluted solution (1 × 10^−5^ M). Figure 1 shows the emission spectra of a 1.5% BBO8-Me n-octanol gel excited at 270 nm and the emission spectra of the gelator in a 1 × 10^−5^ M diluted solution excited at the same wavelengths. The emission spectra are independent of the excitation wavelength either in diluted solution or in the gel state, but there is a slight (4 nm) red shift in the spectrum of the gel indicating the formation of small aggregates. Similar results were observed with all the other gels and solutions, except with BBO6-Et, which does not form gel in any of the solvents studied.

### 2.4. Circular Dichroism

Induced circular dichroism (ICD) is exhibited when the chromophoric moieties of low molecular mass organogelator molecules self-assemble in a certain orientation. ICD spectra of all five 1.5% (*w*/*w*) n-octanol gels were obtained. All gels form chiral aggregates as observed by the ICD spectra (Table 2); however, the dilute solutions are CD silent.

**Figure 2:**
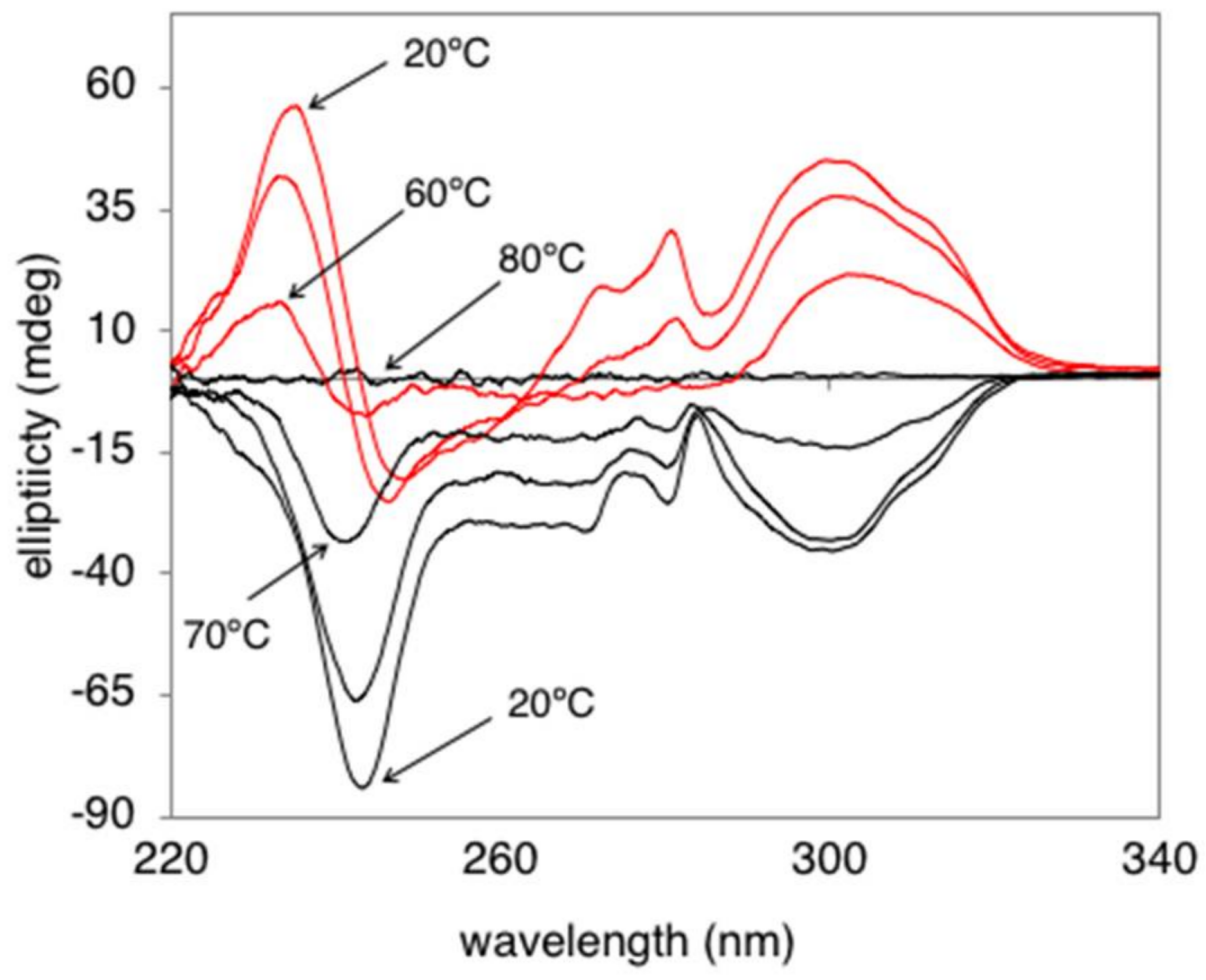
ICD spectra of 1.5% (*w*/*w*) BBO8-Me n-octanol gel-sol transitions (Cycle 1, increasing temperature, black) and sol-gel (Cycle 2, decreasing temperature, red).

The ICD spectrum of a freshly-prepared 1.5% (*w*/*w*) BBO8-Me n-octanol gel shows two significant negative Cotton effects at 244 and 300 nm. Temperature-dependent CD spectroscopy measurements show that the intensity of both CD signals decreases gradually with increasing temperature and eventually disappears at its phase transition temperature (Cycle 1). Since the gelator is not chiral, this is an indication that the ICD signals observed in the gel state originate from the chirality of the aggregate scaffold. CD measurements were also taken while gradually cooling the sol phase (Cycle 2). The ICD spectra show two positive Cotton effects where the intensity of the signals increases with decreasing temperature (Cycle 2, Figure 2).

The thermoreversibility of the gels enables these cycles to be repeated. The signs of the Cotton effects observed are dependent on the physical methods of gel preparation. A series of gel-sol and sol-gel transitions can all yield positive peaks, all negative peaks or both positive and negative peaks. The intensities of the absorption bands depend not only on the concentration, but also on the rate at which the gels are cooled. The slower the rate of cooling, the more ordered the supramolecular structure and the more intense the observed ellipticity. Gels cooled quickly to room temperature or with slight physical disturbance yield weak signals that can be characterized as frailer supramolecular aggregates.

**Figure 3:**
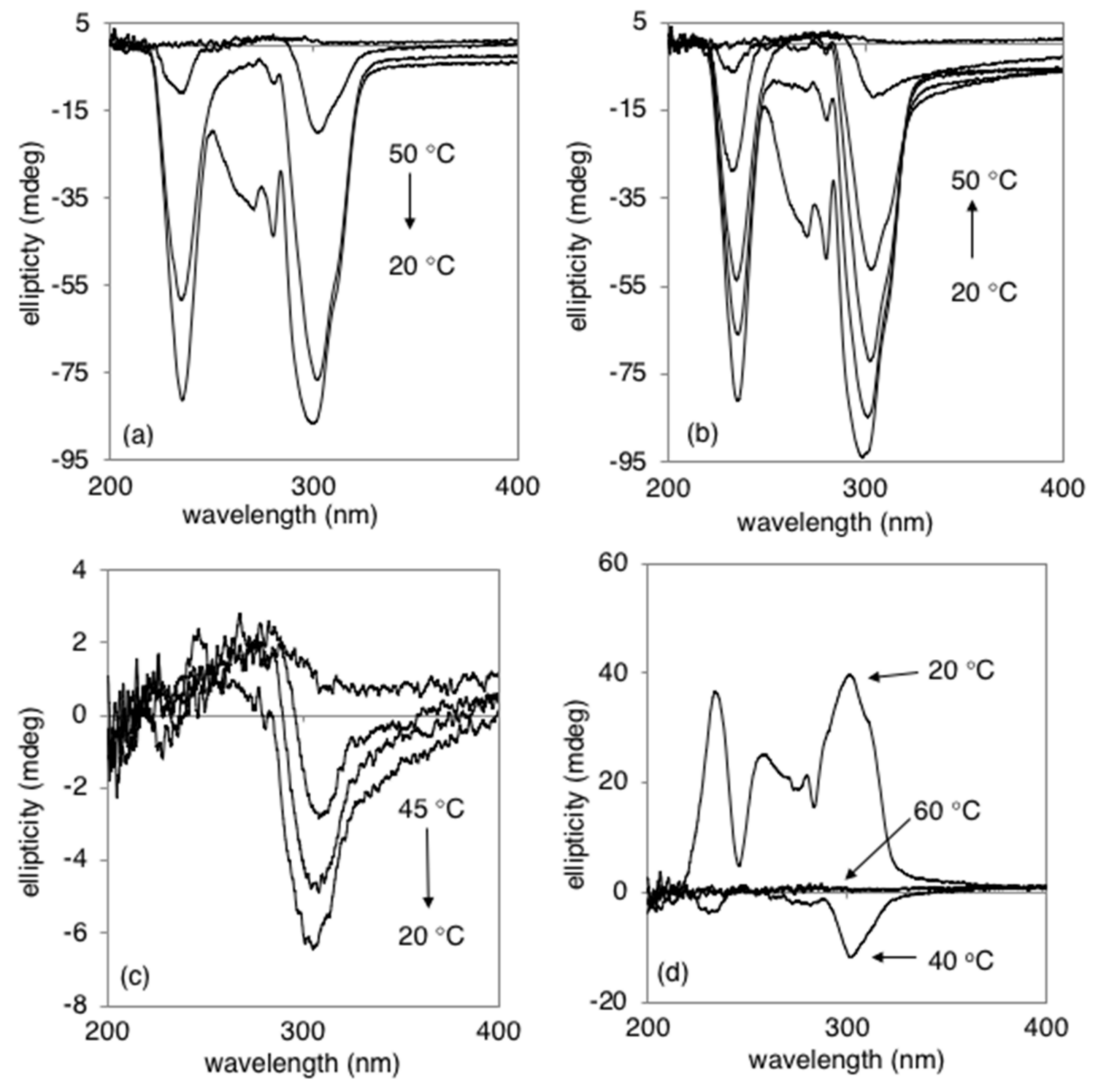
Hysteresis study of 1.5% (*w*/*w*) BBO8-Et n-octanol gel. (**a**) Sol-gel, Cycle 1; (**b**) gel-sol, Cycle 2; (**c**) sol-gel, Cycle 3; (**d**) sol-gel, Cycle 4.

Although these gels are thermoreversible, hysteresis effects and Ostwald ripening factors are observed with an increasing number of heating-cooling cycles. Figure 3 shows the ICD spectra of a series of 1.5% (*w*/*w*) BBO8-Et n-octanol sol-gel and gel-sol transitions. After four consecutive thermoreversible cycles, the structure of the gel begins to degrade as observed by the decreasing ICD signal intensity and changes in the Cotton effects. A freshly-prepared gel was melted, and the gradual cooling from 50–20 °C was monitored. Two negative Cotton effects at 244 and 300 nm started to appear (Figure 3a). The intensity of both absorption peaks increased as the temperature decreased (Cycle 1). The gel obtained was slowly heated from 20–50 °C (Cycle 2), and the two negative Cotton effects gradually decreased (Figure 3b). When this new sol was slowly cooled to room temperature (Cycle 3), only one weak negative absorption peak was observed at around 300 nm, indicating a change in the supramolecular structure of the gel (Figure 3c). Furthermore, after heating this apparently disturbed gel to sol and slowly cooling it back to room temperature, a far different ICD from the initial fresh gel ICD spectra was observed. A single negative Cotton effect was observed at 40 °C, and upon further cooling, two positive Cotton effects were observed at 20 °C (Cycle 4, Figure 3d). The ICD spectral changes observed with consecutive heating-cooling cycles are indicative of gel hysteresis. All fresh gels (one heating-cooling cycle) studied are stable for several months at room temperature; however, the lifetime is dependent on the number of gel-sol transitions. Our results clearly show the sensitivity of the physical methods of preparation on the formation of the gel aggregate scaffold and the subsequent strength of the 3D structure.

### 2.5. Scanning Electron Microscopy Studies

**Figure 4:**
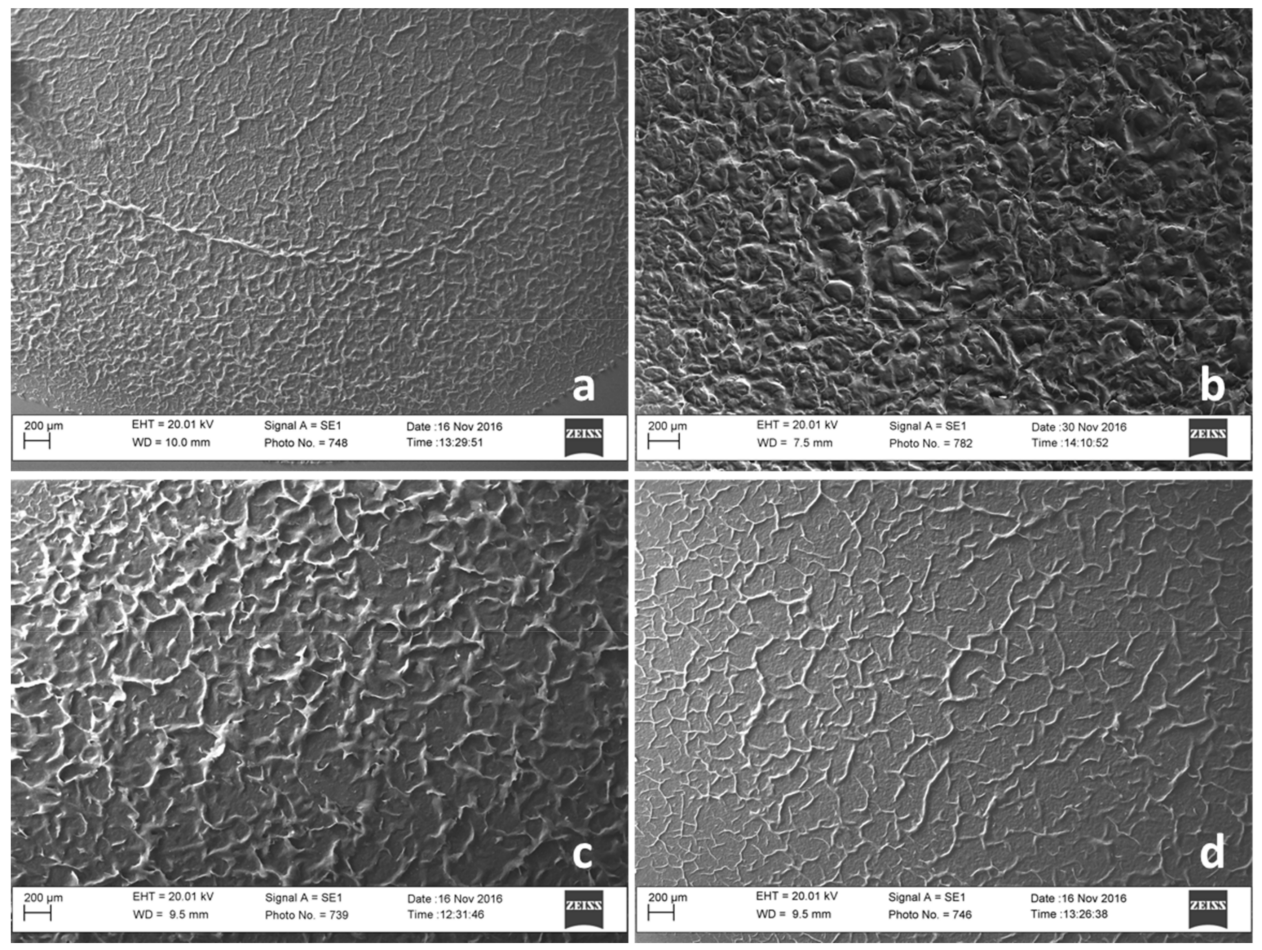
SEM images of 1.5% (*w*/*w*) xerogels. (**a**) BBO6-Me (n-octanol), (**b**) BBO8-Me (n-octanol), (**c**) BBO8-Et (ethanol), (**d**) BBO10-Et (n-octanol).

**Figure 5:**
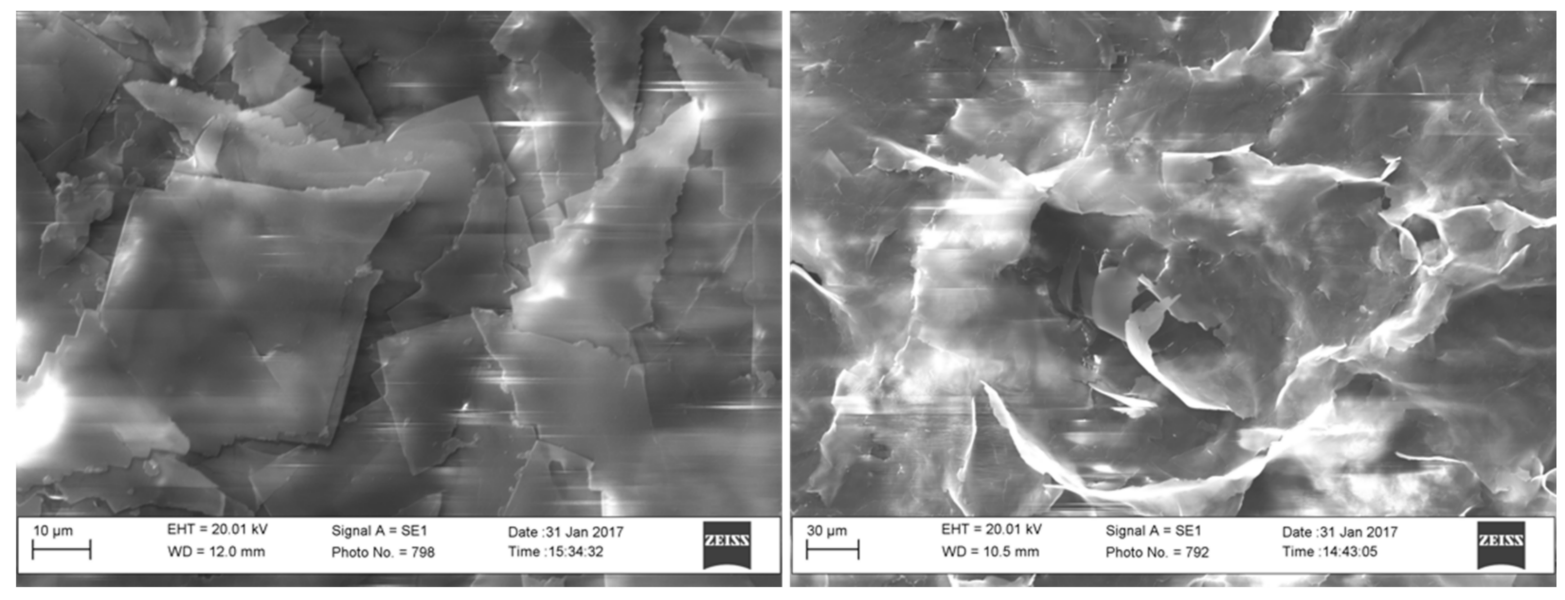
SEM images of BBO6-Me (left) and BBO10-Me (right) xerogels obtained from 1.5% in n-octanol gels.

The morphology of the gels studied is dependent on the number of heating-cooling cycles in a way similar to the ICD hysteresis results previously described and reported by others [27]. For this reason, we report here the results obtained from freshly-prepared gels. SEM images obtained from BBO6-Me, BBO8-Me, BBO8-Et, BBO10-Me and BBO10-Et xerogels acquired from n-octanol, n-butanol and ethanol gels show a similar morphology resembling twisted fibers with a porous network. Figure 4 shows a representative image of each xerogel obtained from the different solvents utilized. Figure 5 shows a magnified view of the BBO6-Me n-octanol xerogel structure. A close look at the fibers reveals the formation of plate-shaped collections resembling dendritic multi-branching tree-like aggregates arranged in a helical fashion; see Figure 5 (left panel). Among all the gels studied, BBO6-Me is the least stable gel as observed by its phase transition temperature and lack of reversibility after two gel-sol transitions. Its lower stability could be attributed to plate formation instead of continuous fiber formation at the nanoscale.

Figure 5 (right panel) shows a closer look at the BBO10-Me n-octanol xerogel where the morphology can clearly be seen as twisted ribbon-like fibers.

### 2.6. X-ray and XRD Diffraction

**Figure 6:**
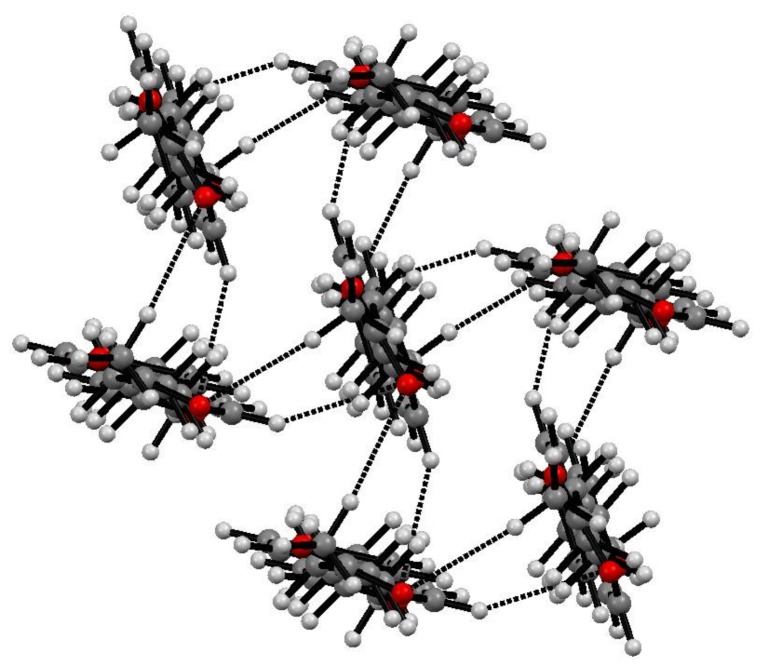
A representation of the way in which the molecules are bundled showing the weak C—H…O hydrogen bonding network as observed in the solid-state structure of the gelator BBO6-Me.

Singles crystals of BBO6-Me and BBO6-Et grown from slow evaporation in CHCl_3_/heptane and CHCl_3_/diethyl ether respectively were obtained. Based on the crystallographic analyses, BBO6-Me and BBO6-Et are isostructural [28]. The data for the reported structures were obtained at 200 K. In order to better compare the unit cell parameters of the solid with the xerogel of BBO6-Me, single-crystal X-ray data were recollected at 293 K. The crystal structure has been deposited at the Cambridge Crystallographic Data Centre and has been assigned the deposition number CCDC 1566046. Figure S1 shows the atom-labeling scheme employed for the symmetry-unique atoms. The unit cell parameters are reported in Table 3. The slight increase in unit cell length as a result of the extra carbon present in the ethyl moiety for BBO6-Et is a motif common to xerogels as shown by the XRD spectra of xerogels. The molecules sit on a crystallographic center of symmetry; thus, one-half of the molecule comprises the asymmetric unit. Figures showing the molecular structure and packing diagram obtained from the room temperature structure determination are available in the Supporting Information. The two phenyl rings are coplanar. The extended structure reveals that each molecule is surrounded by six symmetry-related molecules approximating a hexagonal arrangement. Four of the surrounding molecules are involved in a network of C—H…π and C—H…O interactions with the central molecule and have a molecular centroid separation of 4.94 Å. The remaining two are further removed with a molecular centroid separation of 6.53 Å, the *c*-axis length. As has been previously reported, the interaction energies were found to be −72 kJ/mol and −51.9 kJ/mol, respectively, with the primary components of the interaction being dispersive rather than electrostatic [28]. Interaction energies were calculated using CrystalExplorer17 [29,30,31], which employs the CE-B3LYP/6-31G(d,p) functional/basis set combination and were corrected for basis set superposition energy using the counterpoise method. The bundles of six (see Figure 6) are arranged to form columns with a width corresponding to the length of the *a*-axis. Figure S2 is a packing diagram revealing the columnar nature of the superstructure. The intercolumnar interactions are much weaker (−17.5 kJ/mol) than those within the columns.

**Figure 7:**
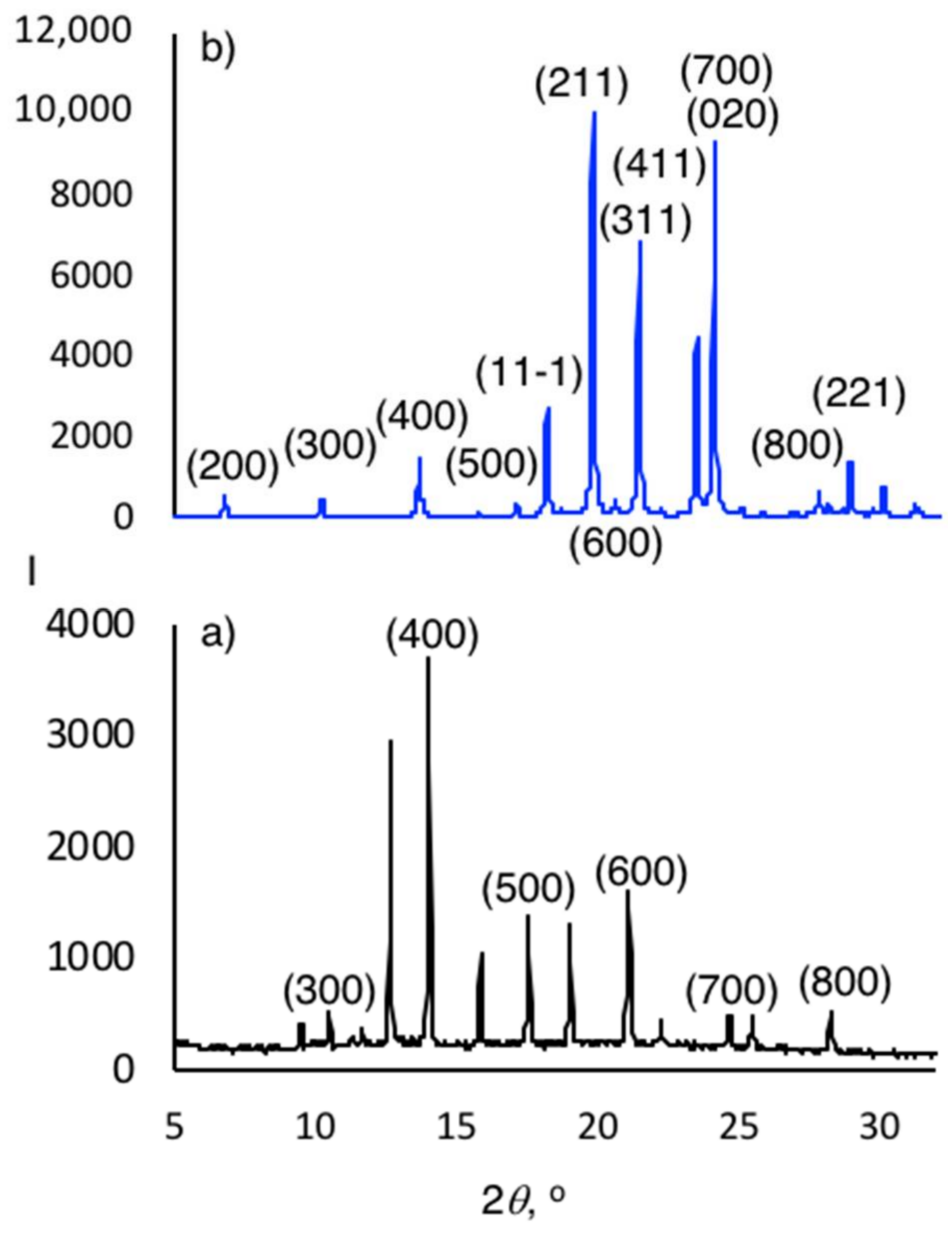
XRD diffractograms (λ = 1.5418 Å): (**a**) BBO6-Me xerogel; (**b**) simulated BBO6-Me based on single-crystal X-ray diffraction results.

The XRD pattern bears a striking similarity to the simulated XRD spectrum based on the single-crystal diffraction study of BBO6-Me. Figure 7 shows the two XRD diffractograms with some of the peaks identified. Of particular interest is the presence of the prominent series of (h00) peaks corresponding to a *d* spacing of 25.17 Å, which is approximately the length of the *a*-axis in the solid state and suggests that the columnar extended structure observed in single crystals is also present in the xerogel.

**Figure 8:**
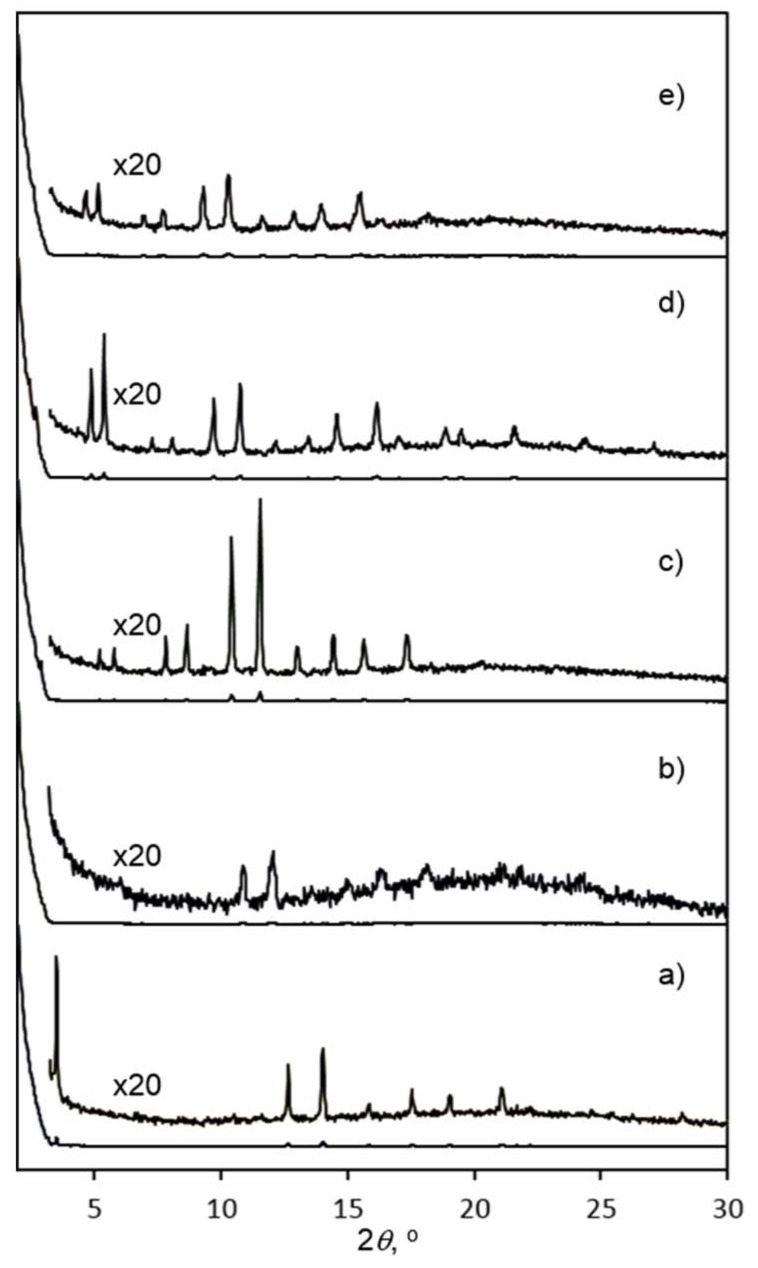
XRD diffractograms (λ = 1.5418 Å) obtained for xerogels. (**a**) BBO6-Me; (**b**) BBO8-Me; (**c**) BBO8-Et; (**d**) BBO10-Me; (**e**) BBO10-Et.

**Figure 9:**
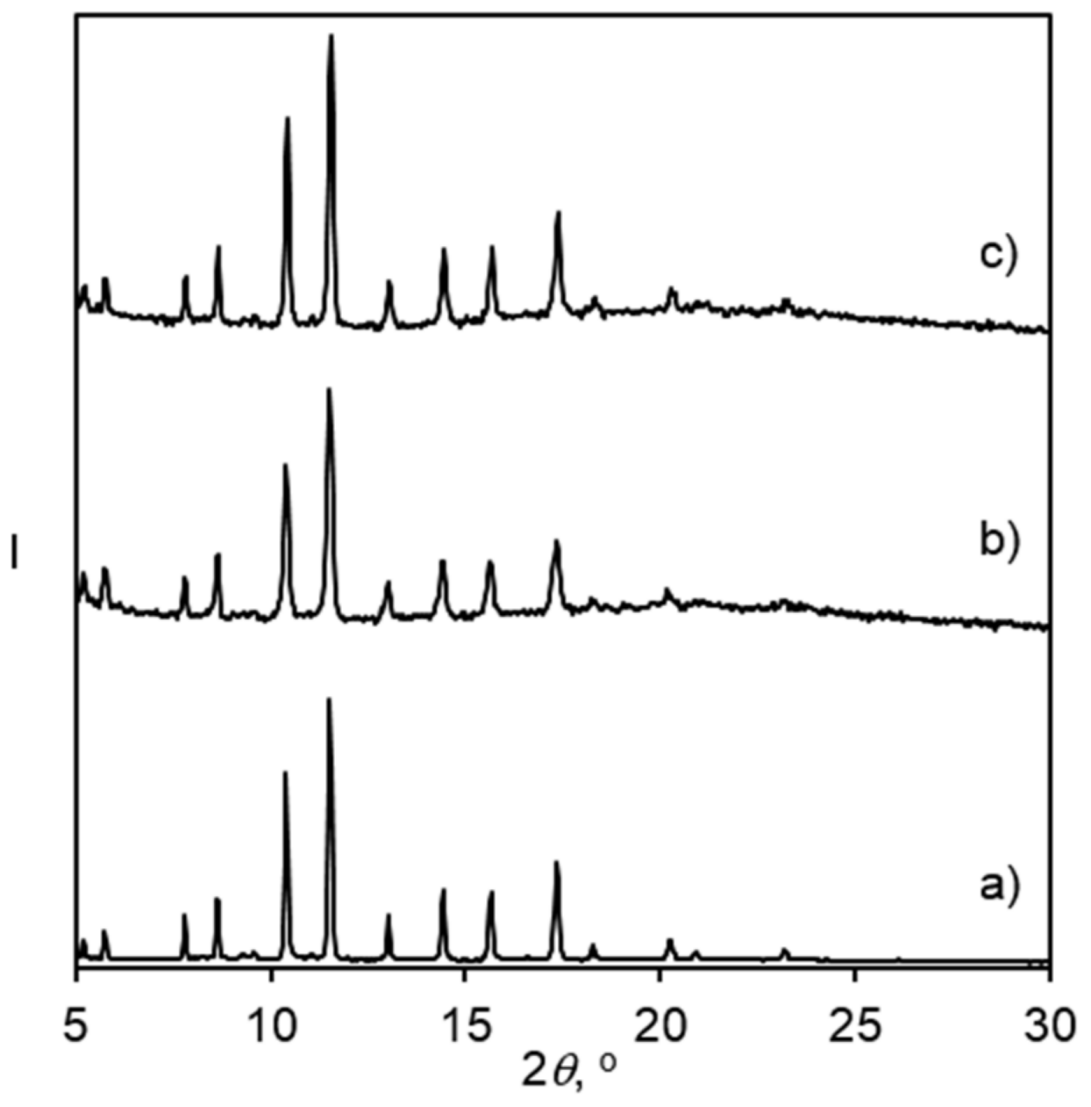
XRD diffractograms (λ = 1.5418 Å) of BBO8-Me xerogels obtained from gels prepared in (**a**) n-octanol, (**b**) n-butanol and (**c**) ethanol.

As seen in Figure 8, the XRD spectra of xerogels from the entire series of BBOn-R gels display similar diffractograms with repeat spacing increasing with chain length. Based on the molecular packing observed for BBO6-Me and BBO6-Et, the xerogels are isostructural with a columnar superstructure. Table 4 shows the 2θ values for the main reflection peaks and the repeat unit spacing as calculated from Bragg’s law, which we interpret to be the column width. The ratios observed are consistent with a lamellar-like structure of the aggregate in the xerogel. Further evidence of the preference for a columnar super-structure is provided by the virtually identical XRD patterns observed for the xerogels of BBO8-Me produced from gels formed in n-octanol, n-butanol and ethanol (see Figure 9).

## 3. Conclusions

Six biphenyl-esters linked by a flexible alkyl chain of varying length were synthesized and their gelation abilities were evaluated in different solvents. The ability of these non-steroidal gelators to form stable and chiral gels shows that the length of the alkyl chain, and therefore the degree of van der Waals interactions, and C—H…π and C—H…O interactions are sufficient driving forces in gel network formation.

All of the gels obtained are thermoreversible, but their stability and the nature of the gel aggregate structure depends on the physical methods of preparation and the number of gel-sol cycles. CD temperature studies clearly show that the sign of the Cotton effects is dependent on gel history. A series of gel-sol and sol-gel transitions yields all positive, all negative or both positive and negative peaks, and peaks can change from negative to positive. The magnitude of the absorbance change depends not only on concentration of the gelator, but also on the rate at which the gel is heated or cooled and the number of sol-gel cycles.

SEM images of BBO6-Me xerogel reveal a platelet aggregate formation arranged in helical fashion, while the morphology of the xerogels obtained from the gelators possessing longer alkyl chain linkers varies from twist ropes to twist ribbon-like structures. The morphology and structure of the xerogels studied are also dependent on the history and preparation of the gel, while the solvent has little to no effect, as seen by SEM and XRD.

The extended structures obtained from single-crystal analysis of BBO6-Me and BBO6-Et gelators reveal that the molecules are bundled in approximately hexagonal sets. The bundles form columns with a width corresponding to the length of the *a*-axis. The XRD pattern obtained from BBO6-Me xerogel is similar to the simulated XRD spectrum based on the single-crystal diffraction study of BBO6-Me. The prominent series of (h00) peaks corresponding to a *d* spacing of 25.17 Å suggests that the columnar extended structure observed in single crystals is also present in the xerogel. Based on XRD analyses, a lamellar packing structure of the gelators is proposed for the gels studied.

## 4. Materials and Methods

### 4.1. Materials and Instrumentation

All of the starting materials and solvents were purchased from commercial suppliers and used as received. Deuterated solvents were purchased from Cambridge Isotope Laboratories. The progress of the reactions and the purity of the final products were monitored by thin layer chromatography (TLC) using TLC plates from J.T. Baker. Ultraviolet spectra were obtained on a Varian Cary-50 Bio UV-visible Spectrophotometer (Agilent, Santa Clara, CA, USA). Steady-state fluorescence spectra were recorded on a Photon Technology International Inc. (Photon Technology International, Birmingham, NJ, USA) QM-40 spectrofluorometer and were not corrected. The CD spectra were obtained on a JASCO J-815 CD spectrometer (Easton, MD, USA). Optical spectroscopy of the gels was performed on samples that were prepared in a 0.1-mm quartz cell. Temperature-dependent CD studies were performed using a JASCO Peltier temperature controller (Easton, MD, USA). Fluorescence spectra were obtained using a front-face detection method. NMR spectra were obtained on an Agilent Advance DPX 400 NMR spectrometer (Santa Clara, CA, USA). The molecular masses of the final products were verified via mass spectrometry at the Lincoln Nebraska Center for Mass Spectrometry (Lincoln, NE, USA). The XRD measurements were conducted using a PANalytical X’Pert Pro diffractometer (Westborough, MA, USA). The XRD pattern was obtained using CuKα radiation with an incident wavelength of 0.154 nm under a voltage of 45 kV and a current of 40 mA. The scan rate was 0.5°/min. The peak positions were identified using the X’Pert high score program. A Zeiss EVO scanning electron microscopy (SEM, Thornwood, NY, USA) was used to obtain images of the xerogels. Samples were prepared by transferring a hot solution of the gel (sol) onto a glass slide then dried for 1 day at room temperature and under vacuum for 1 week. The slide was sputter coated with Au/Pd and imaged using an accelerating voltage of 20 kV. Single-crystal X-ray diffraction studies were performed on a Bruker SMART X2S benchtop diffractometer (Madison, WI, USA).

### 4.2. Gel Preparation and Characterization

The gelation behavior was examined in a number of solvents ranging from apolar to polar. n-Octanol proved to be the best solvent for the gelation of all compounds. Gels were prepared by placing a weighed amount of gelator and solvent in a screw-capped glass vial, which was then heated in a silicon oil bath until all the solid was dissolved. The solution was allowed to cool to room temperature. Two percent gels were prepared using 2 mg of gelator and 98 mg of n-octanol. The formation of a gel was identified when inversion of the vial yielded no movement of the solvent.

Gel absorption, emission and circular dichroism studies were performed in a 0.10-mm demountable quartz cell. A drop of the gel (sol) was spotted onto the slide, and a seal was formed with the addition of the top slide. Temperature measurements were obtained after allowing each gel/sol state to equilibrate for 5 min before a measurement at a new temperature was taken. The phase transition temperature (T_g_) of the gels was determined by placing the vial containing the gel in a thermostatic oil bath and gradually heating until the gel melted into a solution.

### 4.3. Synthetic Details

**Scheme 1:**
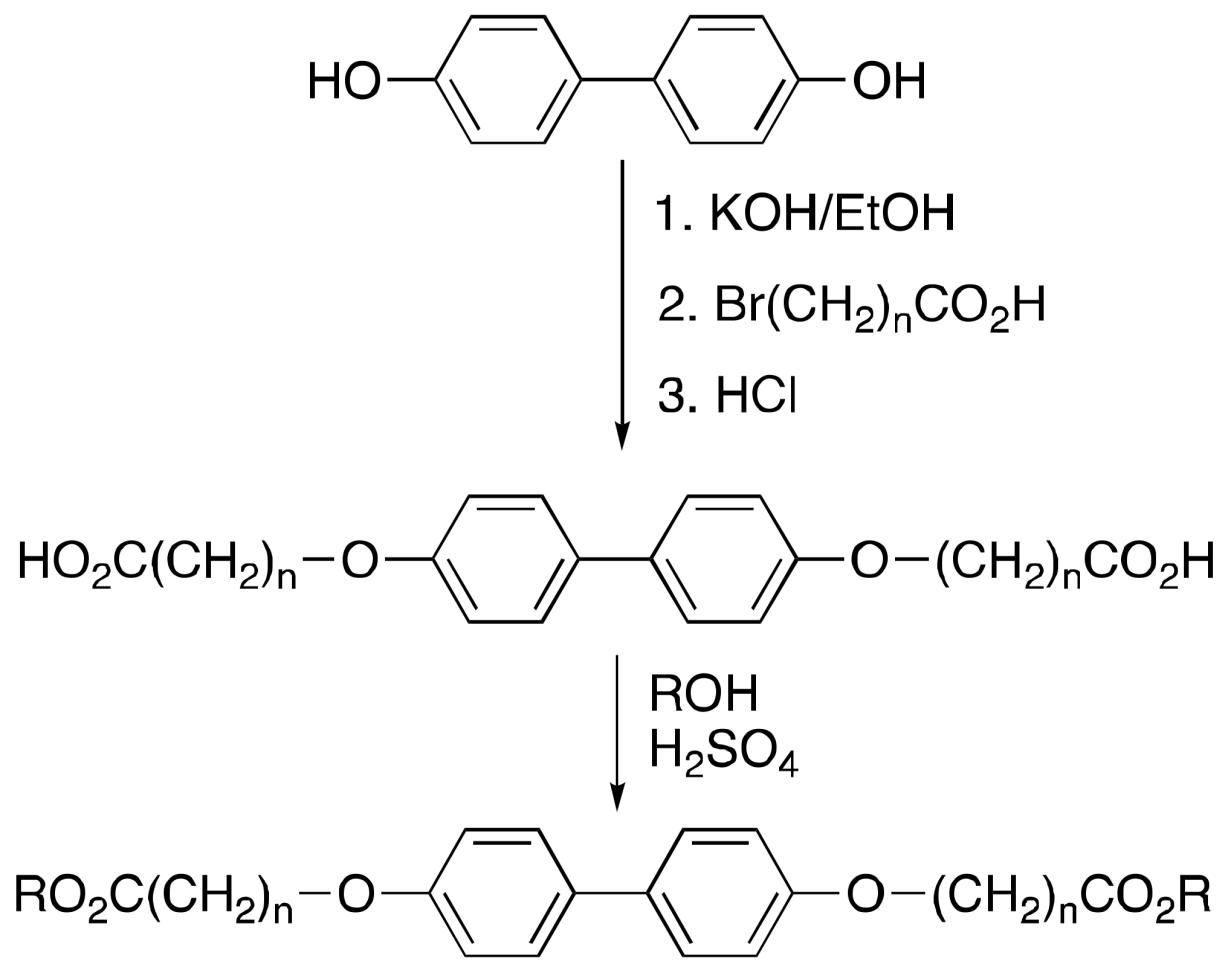
Synthesis of biphenyl gelators. BBO6-Me (*n* = 5, R = Me), BBO6-Et (*n* = 5, R = Et), BBO8-Me (*n* = 7, R = Me), BBO8-Et (*n* = 7, R = Et), BBO10-Me (*n* = 9, R = Me), BBO10-Et (*n* = 9, R = Et).

The synthetic outline used to prepare the gelators is shown in Scheme 1. The acid precursors BBO6A, BBO8A and BBO10A were synthesized by refluxing [1,1′-biphenyl]-4,4′-diol with two equivalents of bromohexanoic acid, bromooctanoic acid and bromodecanoic acid, respectively, and potassium hydroxide in ethanol followed by acidification. All acids were obtained in good percentage yield and their structures analyzed by ^1^H and ^13^C NMR spectroscopy. The corresponding methyl and ethyl esters were obtained by Fisher esterification of the acids with methanol or ethanol under acidic conditions.

#### 4.3.1. Synthesis of Acid Precursors

All acid precursors were prepared following the same procedure as described for the synthesis of BBO8A.

##### 4,4′-biphenoxydioctanoic Acid (BBO8A)

4,4′-biphenol (0.417 g, 2.24 mmol), 8-bromoctanoic acid (1.00 g, 4.48 mmol) and potassium hydroxide (0.754 g, 13.2 mmol) dissolved in minimal amount of water were dissolved in 40 mL of 95% ethanol. The reaction mixture was refluxed for 8 h. After cooling, the solvent was reduced to half by rotary evaporation, and the solid was filtered and dried. The salt was dissolved in ethanol/water and heated to boil. Upon complete dissolution of the salt, 12.5% hydrochloric acid was added until pH ~ 5. The acid was filtered and washed with water. The crude product was filtered and recrystallized from ethanol/water. A 0.494 g, 44% yield of the product was obtained. TLC (acetone/hexane, 1:1 *v*/*v*), R_f_ = 0.72. ^1^H NMR (DMSO): δ (ppm) 12.0 (s, 2H), 8.05 (d, 4H), 7.45 (d, 4H), 4.45 (t, 4H), 2.65 (t, 4H), 1.82 (m, 20H). MS (FAB): calcd. for C_28_H_38_O_6_ 470.60, found 470.2668 with Negative ion ESI (M-H)^−^ 469.3 and (M-2H^+^ Na^+^)^−^ 491.3.

##### 4,4′-biphenoxydihexanoic Acid (BBO6A)

The product was obtained in a 40% yield. TLC (acetone/hexane, 1:1 *v*/*v*), R_f_ = 0.64. ^1^H NMR (DMSO): δ (ppm) 12.02 (s, 2H), 7.49 (d, 4H), 6.95 (d, 4H), 3.99 (t, 4H), 2.34 (t, 4H), 1.82 (t, 4H), 1.71 (m, 4H), 1.52 (m, 4H).

##### 4,4′-biphenoxydidecanoic Acid (BBO10A)

This acid was obtained in a 52% yield. TLC (acetone/hexane, 1:1 *v*/*v*), R_f_ = 0.76. ^1^H NMR (DMSO): δ: (ppm) 11.98 (s, 2H), 7.50 (d, 4H), 6.95 (d, 4H), 3.95 (t, 4H), 2.17 (t, 4H), 1.68 (m, 4H), 1.46–1.25 (m, 24H).

#### 4.3.2. Synthesis of Esters

All methyl and ethyl esters were prepared following the same procedure as described for the synthesis of BBO8Me. The ethyl esters were prepared using ethanol instead of methanol.

##### 4,4′-bis-(7-methyloxycarbonyl heptyloxy) Biphenyl (BBO8-Me)

BBO8A (0.50 g, 1.1 mmol), 50 mL of methanol and several drops of sulfuric acid were refluxed for 4–6 h. The reaction mixture was cooled in an ice bath, and the product was filtered and washed with methanol. A 0.46 g, 92% yield of a white solid was obtained. TLC (CH_2_Cl_2_), R_f_ = 0.54. ^1^H NMR (CDCl_3_): δ (ppm) 7.45 (d, 4H), 6.93 (d, 4H), 3.98 (t, 4H), 3.65 (s, 6H), 2.32 (t, 4H), 1.77 (m, 4H), 1.62 (m, 4H), 1.45 (m, 4H), 1.35 (m, 8H). MS (FAB): calcd. C_30_H_42_O_6_ 498.30, found 498.2981.

##### 4,4′-bis-(5-methyloxycarbonylpentyloxy) Biphenyl (BBO6-Me)

This product was obtained in an 83% yield. TLC (CH_2_Cl_2_), R_f_ = 0.47. ^1^H NMR (CDCl_3_): δ (ppm) 7.47 (d, 4H), 6.95 (d, 4H), 4.00 (t, 4H), 3.66 (s, 6H), 2.38 (t, 4H), 1.82 (m, 4H), 1.72 (m, 4H), 1.55 (m, 4H). MS (FAB): calcd. C_26_H_34_O_6_ 442.24, found 442.2330. Structural determination of BBO6-Me was showed in supplementary files, and table S1 for X-ray crystallography details.

##### 4,4′-bis-(5-ethyloxycarbonylpentyloxy) Biphenyl (BBO6Et)

This product was obtained in a 72% yield. TLC (CH_2_Cl_2_), R_f_ = 0.57. ^1^H NMR (CDCl_3_): δ (ppm) 7.45 (d, 4H, 6.91 (d, 4H), δ 4.13 (q, 4H), δ 3.99 (t, 4H), δ 2.34 (t, 4H), δ 1.82 (m, 4H), δ 1.72 (m, 4H), δ 1.54 (m, 4H), δ 1.26 (t, 6H). MS (FAB): calcd. C_28_H_38_O_6_ 470.27, found 470.2668.

##### 4,4′-bis-(7-ethyloxycarbonyl heptyloxy) Biphenyl (BBO8Et)

The yield of the reaction was a 94% yield. TLC (CH_2_Cl_2_), R_f_ = 0.67. ^1^H NMR (CDCl_3_): δ (ppm) 7.45 (d, 4H), 6.93 (d, 4H), 4.12 (q, 4H), 3.96 (t, 4H), 2.30 (t, 4H), 1.78 (m, 4H), 1.62 (m, 4H), 1.46 (m, 4H), 1.36 (m, 8H), 1.23 (m, 6H). FAB [M]^+^ 526.33.

##### 4,4′-bis-(9-methyloxycarbonylnonyloxy) Biphenyl (BBO10Me)

This product was obtained in a 63% yield. TLC ((CH_2_Cl_2_), R_f_ 0.54. ^1^H NMR (CDCl_3_): δ (ppm) 7.47 (d, 4H), 6.95 (d, 4H), 3.98 (t, 4H), 3.67 (s, 6H), 2.30 (t, 4H), 1.79 (m, 4H), 1.62 (m, 4H), 1.46 (m, 4H), 1.4–1.2 (m, 16H). MS (FAB): calcd. C_34_H_50_O_6_ 554.36, found 554.3601.

##### 4,4′-bis-(9-ethyloxycarbonylnonyloxy) Biphenyl (BBO10Et)

This product was obtained in a 73% yield. TLC (CH_2_Cl_2_), R_f_ 0.60. ^1^H NMR (CDCl_3_): δ (ppm) 7.47 (d, 4H), 6.95 (d, 4H), 4.13 (q, 4H), 3.98 (t, 4H), 2.29 (t, 4H), 1.80 (m, 4H), 1.62 (m, 10H), 1.5–1.25 (m, 20H). MS (FAB): calcd. C_36_H_54_O_6_ 582.39, found 582.3899.

## Figures and Tables

**Table 1:** Gelation studies of biphenyl-ester gelators (1.5%, *w*/*w*). Strong gel (sG), weak gel (wG), gelator insoluble or nearly insoluble at near boiling temperature of the solvent (I) and gelator precipitated upon cooling (P).

Solvent	BBO6-Me (1)	BBO-6Et (2)	BBO8-Me (3)	BBO8-Et (4)	BBO10-Me (5)	BBO10-Et (6)
water	I	I	I	I	I	I
methanol	I	I	I	I	wG	wG
ethanol	wG	P	wG	wG	wG	wG
n-butanol	wG	P	sG	sG	sG	wG
n-hexanol	wG	P	wG	wG	wG	wG
n-octanol	sG	P	sG	sG	sG	sG
hexane	I	I	P	P	P	P
heptane	I	I	P	P	wG	wG

**Table 2:** Gel-sol phase transition temperatures and induced circular dichroism (ICD) absorptions of freshly-prepared 1.5% (*w*/*w*) n-octanol gels.

Gelator	T_g_ (°C)	ICD
BBO6-Me	48–50	(+) 285, (−) 244
BBO8-Me	62–64	(−) 300, (−) 244
BBO8-Et	46–48	(−) 300, (−) 237
BBO10-Me	68–70	(−) 292, (−) 245
BBO10-Et	48–50	(−) 300, (−) 241
BBO8-chol^18^	96–98	

**Table 3:** Structural information for BBO6-Me and BBO6-Et single crystals.

Parameter	BBO6-Me	BBO6-Me [28]	BBO6-Et [28]
Molecular formula	C_26_H_34_O_6_	C_26_H_34_O_6_	C_28_H_38_O_6_
Space group	P2_1_/c	P2_1_/c	P2_1_/c
Temperature, K	293 K	200 K	200 K
Cell lengths, Å	a = 25.98(1)	a = 26.022(9)	a = 26.594(7)
	b = 7.459 (3)	b = 7.410(3)	b = 7.534(2)
	c = 6.518(2)	c = 6.531(2)	c = 6.530(1)
Cell angles (α, β, γ), ^o^	90, 94.37(1), 90	90, 94.38(1), 90	90, 95.872 (8), 90
Cell volume, Å^3^	1259.3(8)	1255.7(7)	1301.6(5)

**Table 4:** Derived BBOn-R xerogel *d* values (*a*-axis length) as calculated from XRD data using Bragg’s law.

Ratio/Gelator		BBO6-Me	BBO8-Me	BBO8-Et	BBO10-Me	BBO10-Et
1:1	2θ	3.51	3.01	2.61	2.44	2.32
	*d* (Å)	25.17	29.31	33.74	36.20	37.95
1:2	2θ			5.25	4.85	4.63
	*d* (Å)			16.82	18.18	19.05
1:3	2θ			7.80	7.27	6.94
	*d* (Å)			11.32	12.4	12.71
1:4	2θ	14.01	12.06	10.41	9.71	9.28
	*d* (Å)	6.31	7.33	8.49	9.10	9.51
1:5	2θ	17.54	15.04	13.01	12.14	11.61
	*d* (Å)	5.05	5.88	6.80	7.28	7.61
1:6	2θ	21.07	18.12	15.63	14.57	13.95
	*d* (Å)	4.21	4.89	5.66	6.07	6.43
1:7	2θ		21.09		17.02	16.31
	*d* (Å)		4.21		5.20	5.43
1:8	2θ	28.23	24.39		19.47	
	*d* (Å)	3.16	3.64		4.55	

## Supplementary Materials

The following are available online at http://www.mdpi.com/2310-2861/4/2/34/s1: Details of single-crystal X-ray data collection and analysis, including: Figure S1: Molecular structure of BBO6-Me showing the atom-labeling scheme of the symmetry-unique atoms, Figure S2: Packing diagram of BBO6-Me looking down the *b*-axis showing the columnar nature of the superstructure, Table S1: X-ray crystallography details. A Crystallographic Information File (CIF) for BBO6-Me is also available.
