# Supplementary material for: Non-Steroidal Biphenyl Gelators: Correlation of Xerogel Structure with Solid-State Structure and Circular Dichroism Spectroscopy
Source: Gels. 2018 Apr 16;4(2):34. doi: 10.3390/gels4020034 (PMC6209265; doi:10.3390/gels4020034)
Supplement: Supplementary file 1 [file gels-04-00034-s001.pdf]

Non-Steroidal Biphenyl Gelators: Correlation of Xerogel Structure with Solid-State Structure and CD Spectroscopy

H. Cristina Geiger[1,*], David K. Geiger[1], William R. Roberts,[1], Dominic L. Morell[1], Paul Huttunen[1], Jennifer L. Schulman[1], Melanie Tran[1] and Dori Farthing[2]

[1]Department of Chemistry, State University of New York College at Geneseo, Geneseo, NY 14454, USA

[2]Department of Geological Sciences, State University of New York College at Geneseo, Geneseo, NY 14454, USA

***Corresponding Author:** H. Cristina Geiger, Department of Chemistry, SUNY-College at Geneseo, Geneseo, New York 14454 cgeiger@geneseo.edu

Table of Contents

Structural determination of BBO6-Me S2

Table S1. X-ray crystallography details S3

Figure S1. Molecular structure and atom-labeling scheme of BBO6-Me S4

Figure S2. Packing diagram of BBO6-Me S4

Structural determination of BBO6-Me

A clear colorless crystal of BBO6-Me with dimensions of 0.10 mm × 0.30 mm × 0.60 mm mounted on a Mitegen Micromount was automatically centered on a Bruker SMART X2S benchtop crystallographic system. The data collection temperature was 27°C. APEX2 software was used for preliminary determination of the unit cell. Determination of integrated intensities and unit cell refinement were performed using SAINT. The integration of the data yielded a total of 6718 reflections to a maximum θ angle of 25.43° (0.83 Å resolution).

The constants for the monoclinic unit cell are a = 25.977(10) Å, b = 7.459(3) Å, c = 6.518(2) Å, β = 94.368(11)°, V = 1259.3(8) $Å^3$. They are based on the refinement of the XYZ-centroids of 1210 reflections above 20.0 I/σ(I) with 2.36° ≤ θ ≤ 21.79°.

Data were corrected for absorption effects with SADABS using the multi-scan technique. The ratio of minimum to maximum apparent transmission is 57.1:100. The average residual for symmetry equivalent reflections is R_{int} = 7.39% and R_σ = 9.57%. XPREP determined the space group to be P 1 21/c 1, with Z = 2 for the formula unit, $C_{26}H_{34}O_6$.

The structure was solved with ShelXS [1,2], and subsequent structure refinements were performed with ShelXL [1,2]. The final anisotropic full-matrix least-squares refinement on F_o^2 with 140 variables converged at R_1 = 9.61% for the observed data and wR_2 = 35.12% for all data. The goodness-of-fit was 1.030. The largest peak on the final difference electron density synthesis was 0.51 $e^-/Å^3$, and the deepest hole was −0.29 $e^-/Å^3$ with an RMS deviation of 0.06 $e^-/Å^3$. On the basis of the final model, the calculated density is 1.167 g/cm^3 and F(000) = 476. See Table S1 for other experimental details. The crystal structure has been deposited at the Cambridge Crystallographic Data Centre and has been assigned the deposition number CCDC 1566046.

Table S1. X-ray crystallography details.

Crystal data	
$C_{26}H_{34}O_6$	V = 1259.3 (8) $Å^3$
Mr = 442.53	Z = 2
Monoclinic, $P2_1/c$	Mo $K\alpha$ radiation
a = 25.977 (10) Å	μ = 0.08 mm^{-1}
b = 7.459 (3) Å	T = 300 K
c = 6.518 (2) Å	0.60 × 0.30 × 0.10 mm
β = 94.368 (11)°	
Data collection	
Bruker SMART X2S benchtop diffractometer	2291 independent reflections
Absorption correction: multi-scan SADABS2016/2[1] - Bruker AXS area detector scaling and absorption correction [1]	1061 reflections with $I > 2\sigma(I)$
T_{min} = 0.57, T_{max} = 0.99	R_{int} = 0.074
6718 measured reflections	θ_{max} = 25.4°
Refinement	
R[F2 > 2σ(F2)] = 0.096	0 restraints
wR(F2) = 0.351	H-atom parameters constrained
S = 1.03	$\Delta\rho_{max}$ = 0.51 e $Å^{-3}$
2291 reflections	$\Delta\rho_{min}$ = −0.29 e $Å^{-3}$
140 parameters	
Data collection: APEX2 [1]; cell refinement: SAINT V8.34A [1]; data reduction: SAINT V8 [1]; program(s) used to solve structure: XT, Version 2014/5 [2]; program(s) used to refine structure: SHELXL2014/7 [2]; molecular graphics: PLATON [3].	

1. Bruker **2013**. *APEX2*, *SAINT*, *SADABS*, and XSHELL, Bruker AXS Inc., Madison, Wisconsin, USA.
2. Sheldrick, G. M., SHELXT-Integrated space-group and crystal-structure determination. *Acta Cryst.* **2015**, *A71*, 3–8.
3. Spek, A. L., Structure validation in chemical crystallography, *Acta Cryst.* **2009**, *D65*, 148–155.

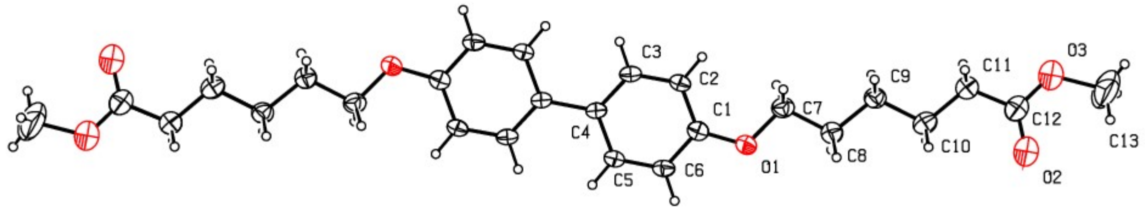

Figure S1. Molecular structure of BBO6-Me showing the atom-labeling scheme of the symmetry-unique atoms. Non-hydrogen anisotropic displacement parameters are drawn at the 30% probability level.

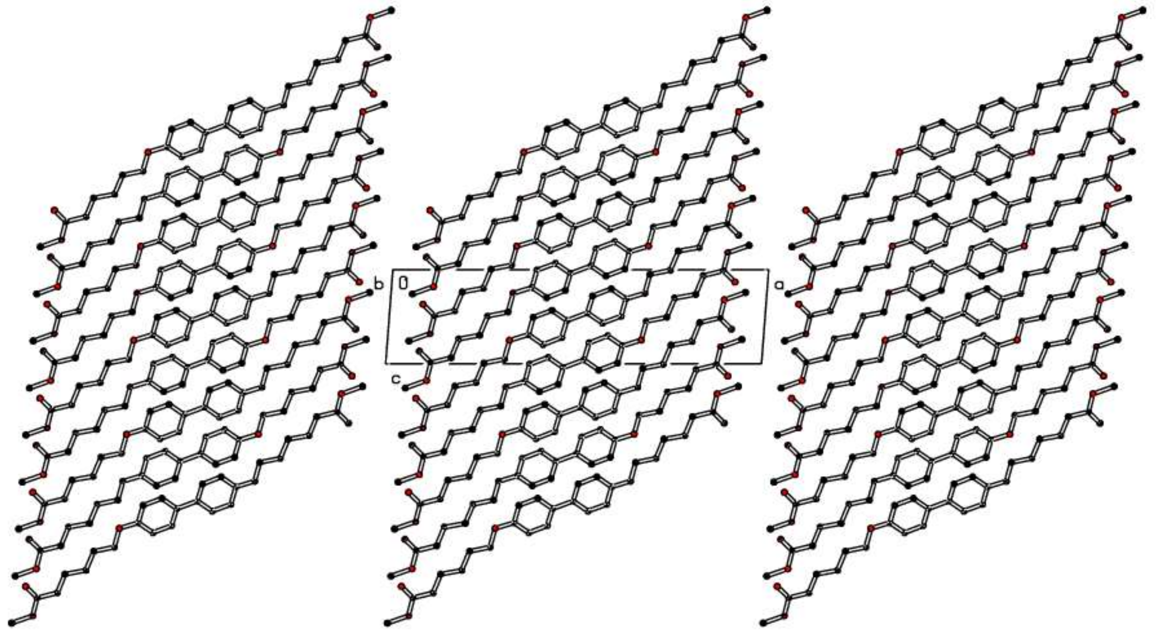

Figure S2. Packing diagram of BBO6-Me looking down the *b*-axis showing the columnar nature of the superstructure.
